# A plasma‐derived exosomal microRNA signature by small RNA sequencing for early detection of postmenopausal osteoporosis

**DOI:** 10.1002/ctm2.1637

**Published:** 2024-04-01

**Authors:** Pan Gao, Sijia Li, Zhanying Dong, Yonglun Luo, Xiuqing Zhang, Linbo Han, Songlin Peng, Jianliang Shen, Fengping Xu, Zaian Deng

**Affiliations:** ^1^ College of Life Sciences University of Chinese Academy of Sciences Beijing China; ^2^ BGI Cell Shenzhen China; ^3^ BGI Research Shenzhen China; ^4^ College of Health Science and Environmental Engineering Shenzhen Technology University Shenzhen Guangdong China; ^5^ Department of Spine Surgery Shenzhen People's Hospital The First Affiliated Hospital Southern University of Science and Technology Shenzhen Guangdong China; ^6^ National Engineering Research Center of Ophthalmology and Optometry Eye Hospital Wenzhou Medical University Wenzhou Zhejiang P. R. China; ^7^ Zhejiang Engineering Research Center for Tissue Repair Materials Wenzhou Institute Wenzhou China; ^8^ University of Chinese Academy of Sciences Wenzhou Zhejiang P. R. China

Dear Editor,

In this study, we revealed specific plasma‐derived exosomal microRNA (exo‐miRNA) signatures associated with postmenopausal osteoporosis (PMOP) and evaluated their potential as biomarkers for early PMOP detection. Our work offered a facile strategy for large‐scale and early PMOP detection, providing valuable insights into the relationship between plasma‐derived exo‐miRNA profiles and the progression of PMOP.

Although the diagnosis of osteoporosis currently mainly relies on imaging techniques, research on using blood markers for osteoporosis diagnosis is ongoing.^1^ As we know, blood testing presents various advantages, such as being more convenient, cost‐effective, capable of detecting disease in early‐stage and suitable for large‐population test.^2^ Several studies have found that plasma exo‐miRNAs can serve as diagnostic biomarkers for PMOP.^3^, ^4^ However, most studies excluded individuals with osteopenia (OPNA), early‐stage of PMOP,^5^ which might impede understanding the roles of exo‐miRNAs in the onset of PMOP and the identification of early detection biomarkers for PMOP.

A total of 272 postmenopausal women volunteers were included in this study, including PMOP (*n* = 60), OPNA (*n* = 160) and healthy controls (CTL, *n* = 52) (Figure S1). None of the participants had claimed a history of diabetes or hepatitis B. Each sample group was split into two subgroups: one for exo‐miRNA profiling and biomarker discovery, and the other for the validation cohort. The age differences in the three groups were considered and controlled during the data analysis. The workflow diagram of this study is illustrated in Figure S2. The clinical information and characteristics of all participants were presented in Tables S1 and S2.

The workflow for isolating and analyzing plasma‐derived exo‐miRNA was described in Figure 1A and Supplementary information. Results from Western blot, transmission electron microscope (TEM) and nano‐flow cytometry (nFCM) analyses confirmed the successful isolation of exosomes (Figure S3). Small RNA (sRNA) sequencing revealed various sRNA biotypes in plasma‐derived exosomes, with miRNA being the most abundant (Figure 1B, Table S3). A total of 487 exo‐miRNAs were selected for miRNA expression profiling analysis (Table S4), and the most abundant fifteen miRNAs were presented in Figure 1C. Comparative analysis identified 9, 17 and 53 differentially expressed exo‐miRNAs (DE‐exo‐miRNAs) in the PMOP vs. OPNA, PMOP vs. CTL and OPNA vs. CTL comparisons, respectively (Figure 1D–F, Table S5). Hierarchical clustering based on DE‐exo‐miRNAs resulted in two distinct sample clusters: one primarily containing PMOP and OPNA samples, and the other mainly containing CTL samples (Figure 1G). The results suggested that the plasma‐derived exo‐miRNAs expression profiles were associated with PMOP and OPNA, making them a promising resource for developing biomarkers to detect PMOP and OPNA.

**FIGURE 1:**
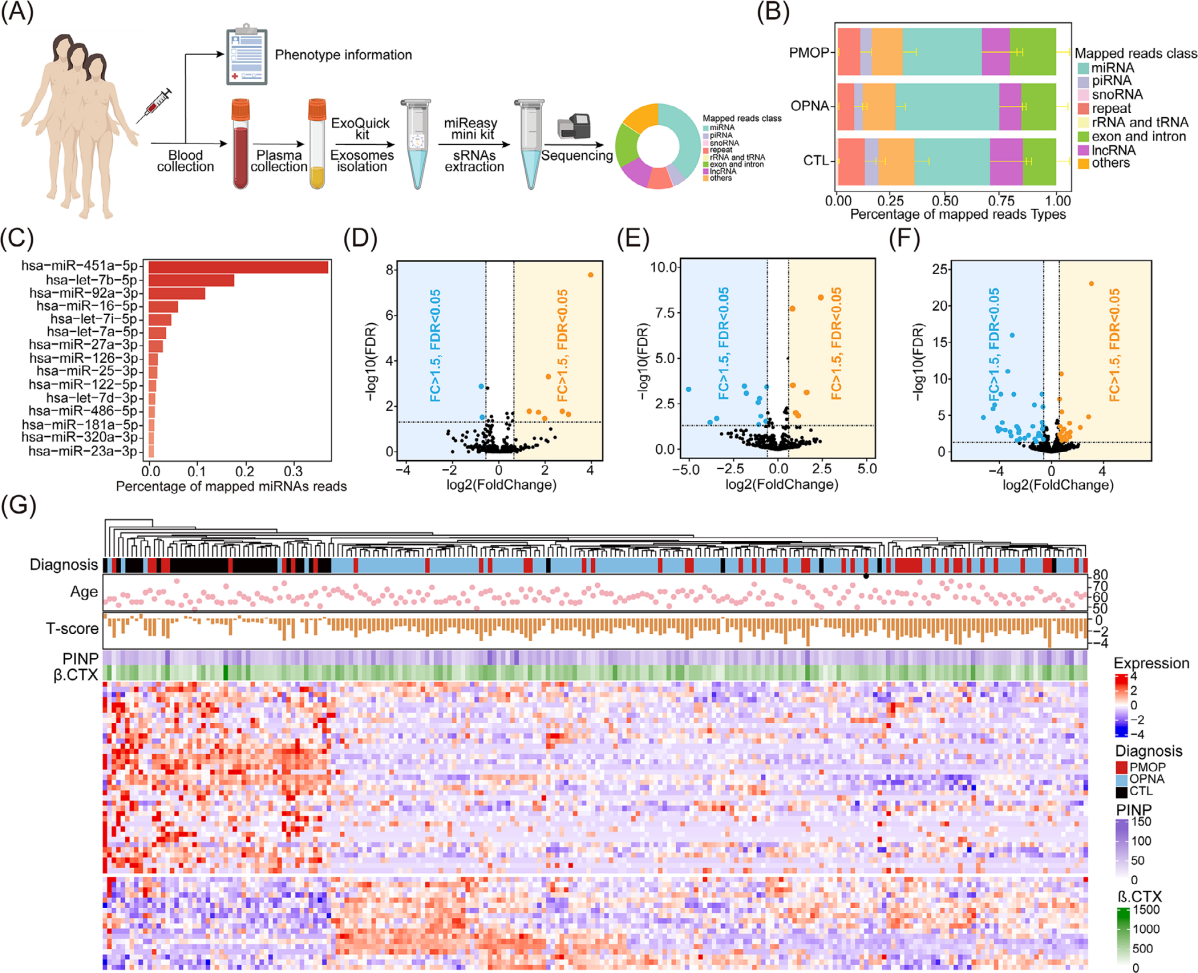
Differential plasma‐derived exosomal miRNAs expression profiles in PMOP, OPNA and CTL groups. (A) Workflow of sample collection. (B) Percentage of all clean reads mapped to various ncRNA databases in the three groups. (C) Top 15 highest abundance plasma‐derived exosomal miRNAs in postmenopausal women. Volcano plots of DE‐miRNAs in the PMOP vs. OPNA (D), the PMOP vs. CTL (E) and the OPNA vs. CTL (F), respectively. (G) Hierarchical clustering of DE‐miRNAs in PMOP, OPNA and CTL groups.

Venn diagrams illustrated the overlap of DE‐miRNAs exhibiting the same change trends in both OPNA and PMOP groups compared to the control groups, as shown in Figure 2A and Table S6. Regulatory networks, constructed using DE‐miRNAs and their target genes, reveal that DE‐miRNAs could target multiple genes and some genes could be targeted by two or three DE‐miRNAs (Figure 2B, C). The GO and KEGG analyses were performed based on these target genes (Table S7–S10), unveiling significantly enriched biological process terms and pathways associated with bone metabolism, including ossification, osteoblast differentiation and negative regulation of osteoclast differentiation, etc. (Figure 2D–G). These results suggested that the concentration of circulating exo‐miRNA may reflect and/or regulate the progression of PMOP and OPNA by influencing multiple signalling pathways.

**FIGURE 2:**
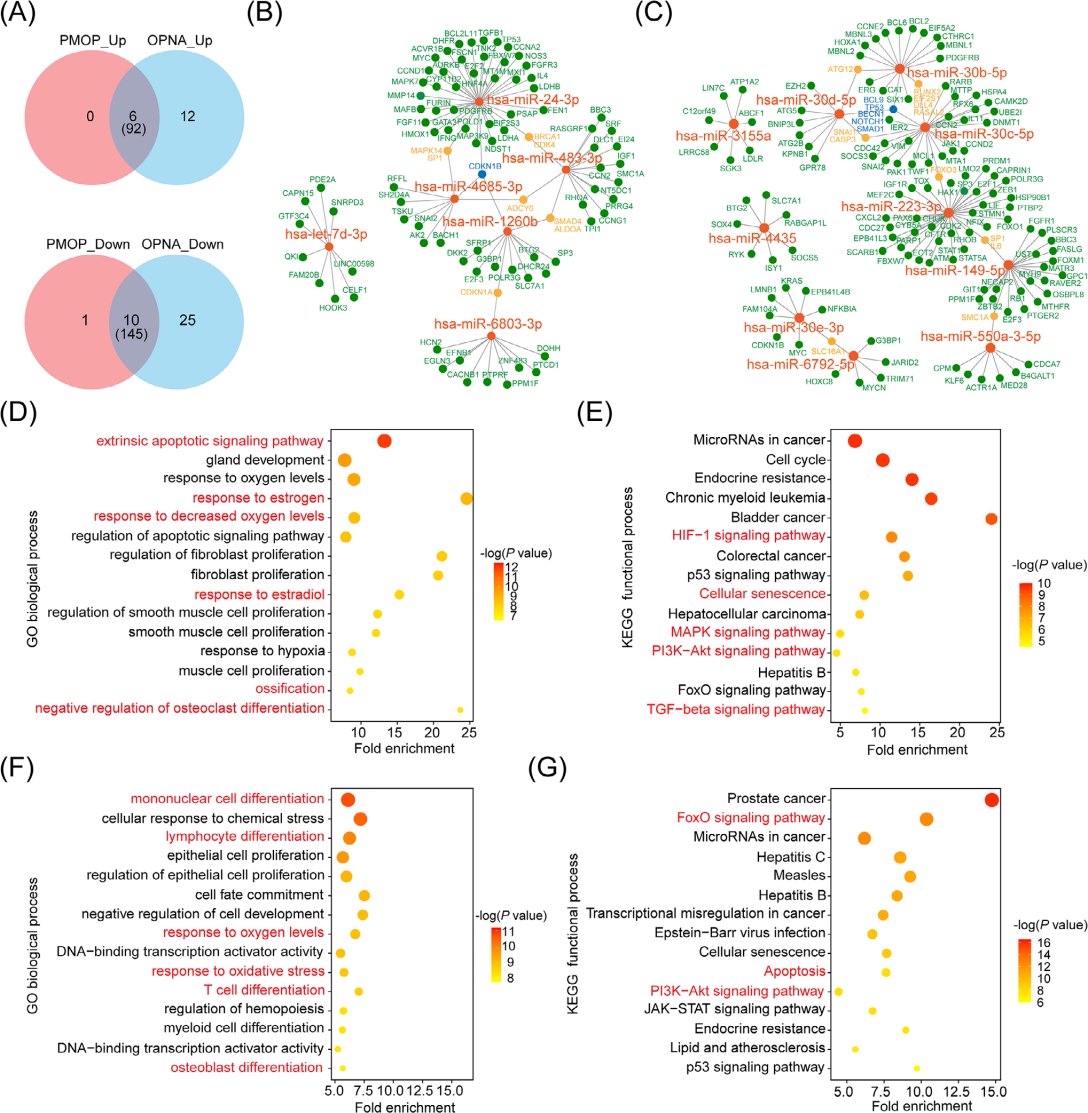
Target gene prediction and functional enrichment analysis. (A) Venn diagrams show the number of overlapping differentially expressed exosomal miRNAs and its target genes between groups. Interaction networks of the overlapping upregulated (B) and downregulated (C) exosomal miRNAs and target genes, red represent miRNAs, green represent genes targeted by one miRNA, orange represent genes targeted by two miRNAs and blue represent genes targeted by three or more miRNAs. GO enrichment analysis (D) and KEGG enrichment analysis (E) for target genes of the overlapping upregulated exosomal miRNAs. GO enrichment analysis (F) and KEGG enrichment analysis (G) for target genes of the overlapping downregulated exosomal miRNAs, red represent some noteworthy signaling pathways.

The minimized exo‐miRNA signatures for distinguishing PMOP from CTL were determined using lasso‐logistic regression in the discovery cohort, identifying hsa‐let‐7d‐3p, hsa‐miR‐24‐3p and hsa‐miR‐550a‐3‐5p as potential biomarkers for PMOP detection (Figure S4). A previous study noted that the miR‐24‐3p level was significantly higher in serum and bone tissues,^6^ and could negatively regulate osteogenic differentiation by targeting Tcf‐1,^7^ which is consistent with our findings. Importantly, this is the first report that let‐7d‐3p and miR‐550a‐3‐5p may be related to PMOP.

In the discovery cohort, receiver operating characteristic (ROC) analyses revealed the area under curve (AUC) values of the three exo‐miRNAs were .84, .80 and .82, respectively (Figure 3A–C). When combined as a panel, these three exo‐miRNAs significantly enhanced discrimination efficiency for PMOP, yielding an AUC of .91 (Figure 3D, Table S11). Notably, the exo‐miRNA panel exhibited superior discrimination ability compared to two bone turnover markers (BTMs), procollagen I N‐Terminal propeptide (PINP) and β isomer of C‐terminal telopeptide of type I (β.CTX), which are a potential biomarker for osteoporosis.^8^, ^9^ Additionally, the robustness of this exo‐miRNA panel's performance was confirmed in the validation cohort (Figure 3E, Table S12). Importantly, ROC analysis conducted in the two OPNA cohorts revealed significant potential in using these three exo‐miRNAs individually and as a panel to detect OPNA patients from CTL (Figure S5A–C, Figure 3F, G, Table S12), suggest that the plasma‐derived exo‐miRNA signature could also effectively distinguish early‐stage PMOP from CTL.

**FIGURE 3:**
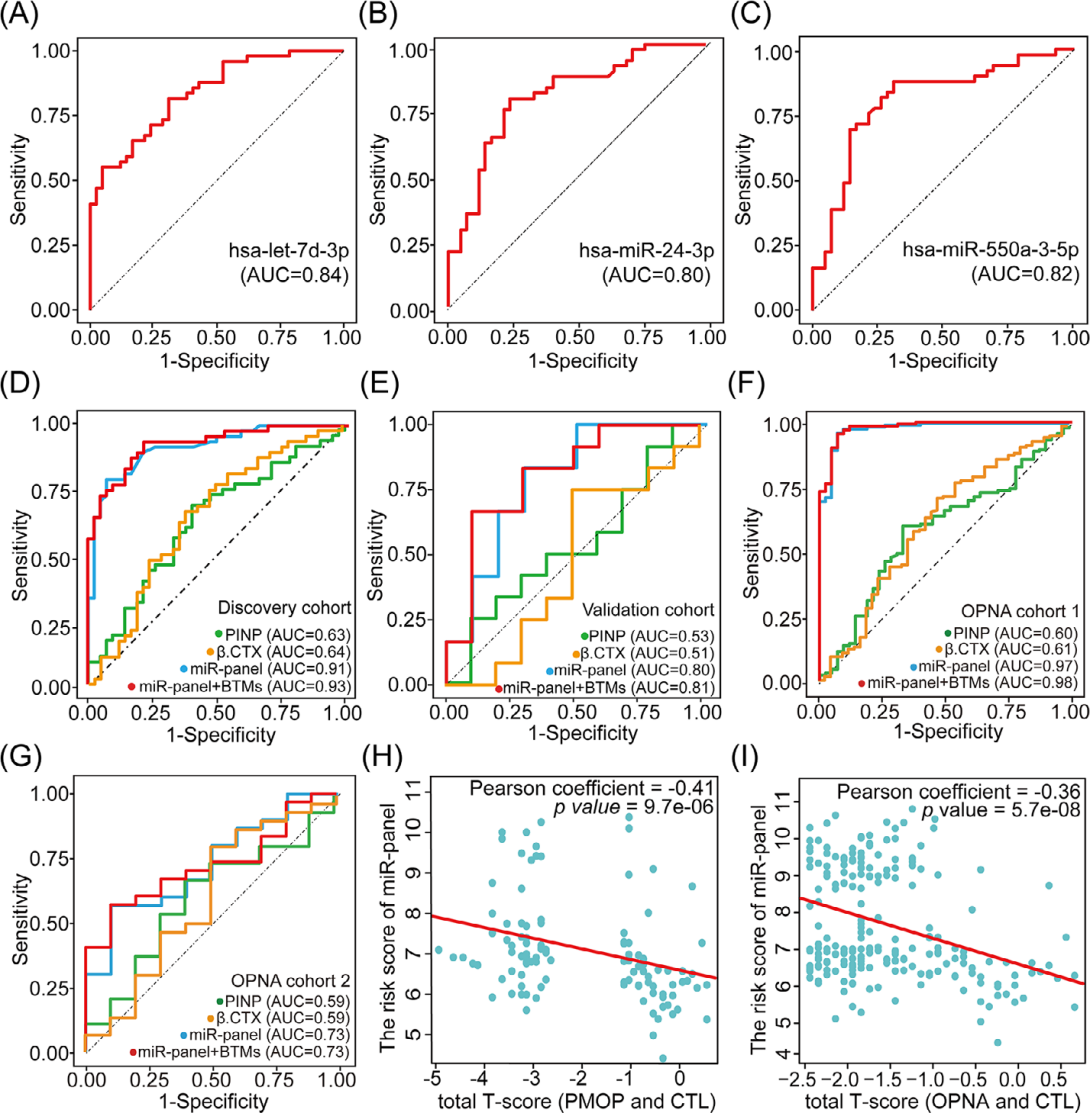
Performances of the three exosomal miRNAs as potential biomarkers for early PMOP detection. The ROC curves of hsa‐let‐7d‐3p (A), hsa‐miR‐24‐3p (B) and hsa‐miR‐550a‐5p (C) in the discovery cohort, respectively. AUC curves of multiple risk models in the discovery cohort (D) and the validation cohort (E). Bone turnover markers (BTMs) include the PINP and β.CTX. AUC curves of multiple risk models in the OPNA cohort one (F) and the OPNA cohort two (G). (H) Correlation between the total T‐score and the risk score of exosomal miRNA panel in all PMOP and CTL samples. (I) Correlation between total T‐score and the risk score of exosomal miRNA panel in all OPNA and CTL samples.

Interestingly, the risk score calculated based on the expression levels of the three exo‐miRNAs^10^ displayed a moderate negative correlation with the total T‐score (Figure 3H and I), as detailed in Table S13. These findings implied that the three exo‐miRNAs may not only serve as detection biomarkers but also act as functional molecules influencing the progression of PMOP and OPNA. Additionally, the moderate expression levels of these three exosomal miRNAs from blood, mesenchymal stem cells and fibroblasts, as indicated by public databases, suggesting that these miRNAs may potentially originate from these cellular sources (Figure S6). However, further in vitro and in vivo studies are essential to elucidate the molecular mechanisms of these exo‐miRNAs in PMOP and OPNA progression.

The relative expression levels of the three exo‐miRNAs in the three groups were shown in Figure S7A–F, successfully validated by RT‐qPCR (Figure 4A–C). It further affirmed the reliability of the proposed exo‐miRNAs as biomarkers for PMOP detection.

**FIGURE 4:**
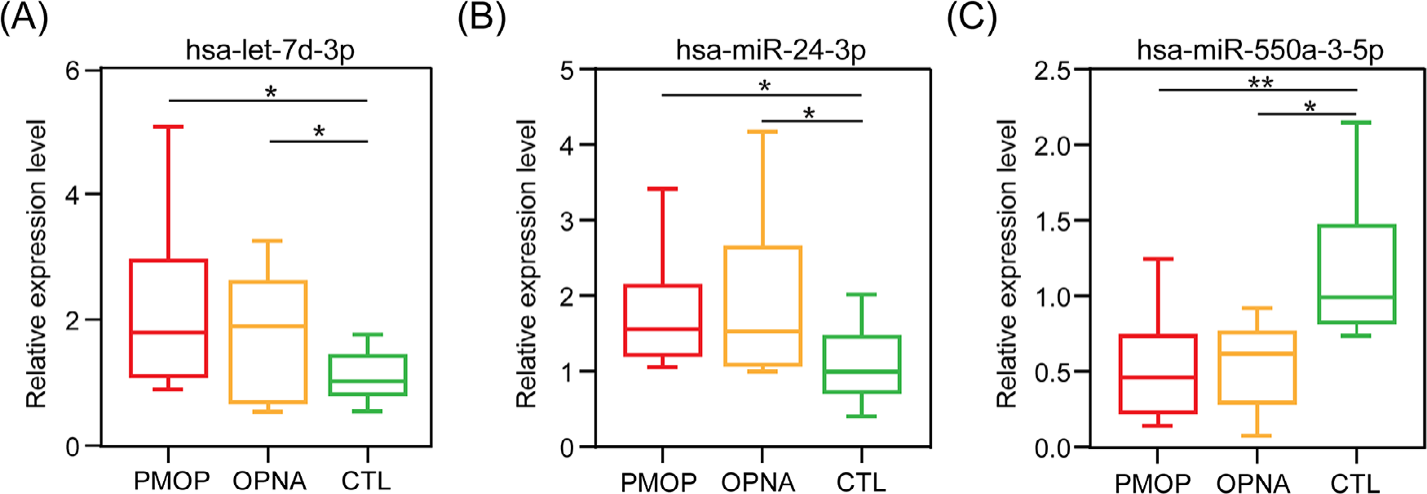
Box plots represent relative expression levels of hsa‐let‐7d‐3p (A), hsa‐miR‐24‐3p (B) and hsa‐miR‐550a‐3‐5p (C) in plasma‐derived exosomes from PMOP, OPNA and CTL using RT‐qPCR. (*p < 0.05, **p < 0.01, ***p < 0.001).

In conclusion, we performed large‐scale plasma‐derived exo‐miRNA profiling in PMOP, OPNA and CTL individuals, identifying a robust plasma‐derived exo‐miRNA signature with significant discriminative power for detecting early PMOP. Our findings proposed a minimally invasive strategy for the early and large‐scale detection of PMOP, which has great potential to be promoted into clinical practice.

## AUTHOR CONTRIBUTIONS

Zaian Deng, Fengping Xu, Jianliang Shen, Songlin Peng and Yonglun Luo conceived the study. Pan Gao, Sijia Li and Zhanying Dong contributed to the data curation and investigation. Pan Gao, Sijia Li and Zaian Deng did the data analysis and verified the results. Pan Gao was responsible for data visualization and wrote the original manuscript draft. Zaian Deng, Fengping Xu, Jianliang Shen, Linbo Han and Xiuqing Zhang reviewed and edited the manuscript.

## CONFLICT OF INTEREST STATEMENT

The authors declare no conflict of interest.

## FUNDING INFORMATION

This research was supported by the Science, Technology, and Innovation Commission of Shenzhen Municipality under grant no. JCYJ20160301151248779 and grant no. JCYJ2020 0109150410232, Basic and Applied Basic Research Foundation of Guangdong Province (grant no. 2019B1515120034), Natural Science Foundation of Top Talent of SZTU (grant no. 2020109), The Common University Innovation Team Project of Guangdong (grant no. 2021KCXTD041).

## ETHICS APPROVAL AND CONSENT TO PARTICIPATE

This study was approved by the Shenzhen People's Hospital Ethics Committee and BGI Ethics Committee (BGI‐IRB 17081‐T2) and each participant signed informed consent.

## Supporting information

Supporting Information

Supporting Information

Supporting Information

Supporting Information

Supporting Information

Supporting Information

Supporting Information

Supporting Information

Supporting Information

Supporting Information

Supporting Information

Supporting Information

Supporting Information

Supporting Information

Supporting Information

Supporting Information

Supporting Information

Supporting Information

Supporting Information

Supporting Information

Supporting Information

Supporting Information

## Data Availability

All data that support the findings of this study are available within the paper and its supplementary material.

## References

[1] Shetty S , John B , Mohan S , Paul TV . Vertebral fracture assessment by dual‐energy X‐ray absorptiometry along with bone mineral density in the evaluation of postmenopausal osteoporosis. Arch Osteoporos. 2020;15(1):25. 32095943 10.1007/s11657-020-0688-9

[2] Williams S , Khan L , Licata AA . DXA and clinical challenges of fracture risk assessment in primary care. Cleve Clin J Med. 2021;88(11):615‐622. 34728487 10.3949/ccjm.88a.20199

[3] Li D , Liu J , Guo B , et al. Osteoclast‐derived exosomal miR‐214‐3p inhibits osteoblastic bone formation. Nat Commun. 2016;7(1):10872. 26947250 10.1038/ncomms10872 PMC4786676

[4] Shi H , Jiang X , Xu C , Cheng Q . MicroRNAs in serum exosomes as circulating biomarkers for postmenopausal osteoporosis. Front Endocrinol (Lausanne). 2022;13:819056. 35360081 10.3389/fendo.2022.819056 PMC8960856

[5] Bala Y , Zebaze R , Ghasem‐Zadeh A , et al. Cortical porosity identifies women with osteopenia at increased risk for forearm fractures. J Bone Miner Res. 2014;29(6):1356‐1362. 24519558 10.1002/jbmr.2167 PMC4156822

[6] Seeliger C , Karpinski K , Haug AT , et al. Five freely circulating miRNAs and bone tissue miRNAs are associated with osteoporotic fractures. J Bone Miner Res. 2014;29(8):1715‐1717. 24431276 10.1002/jbmr.2175

[7] Zhao W , Wu C , Dong Y , et al. MicroRNA‐24 regulates osteogenic differentiation via targeting T‐cell factor‐1. Int J Mol Sci. 2015;16(5):11699‐11712. 26006243 10.3390/ijms160511699 PMC4463725

[8] Krege JH , Lane NE , Harris JM , Miller PD . PINP as a biological response marker during teriparatide treatment for osteoporosis. Osteoporos Int. 2014;25(9):2159‐2171. 24599274 10.1007/s00198-014-2646-0 PMC4134485

[9] Gillett MJ , Vasikaran SD , Inderjeeth CA . The role of PINP in diagnosis and management of metabolic bone disease. Clin Biochem Rev. 2021;42(1):3‐10. 34305208 10.33176/AACB-20-0001 PMC8252919

[10] Lv W , He X , Wang Y , et al. A novel immune score model predicting the prognosis and immunotherapy response of breast cancer. Sci Rep. 2021;13:6403. 10.1038/s41598-023-31153-2 PMC10115816 37076508

