# Supplementary material for: A plasma‐derived exosomal microRNA signature by small RNA sequencing for early detection of postmenopausal osteoporosis
Source: Clin Transl Med. 2024 Apr 1;14(4):e1637. doi: 10.1002/ctm2.1637 (PMC10983017; doi:10.1002/ctm2.1637)
Supplement: Supplementary file 1 — Supporting Information [file CTM2-14-e1637-s020.docx]

**Supporting Information**

**Participants and sample collection**

In this study, all participants with PMOP, OPNA and CTL were determined according to the clinical diagnostic criteria of T-score (T-score≤-2.5 for PMOP, -2.5＜T-score＜-1.0 for OPNA, T-score≥-1.0 for CTL). Peripheral blood samples (5 mL) were collected from every individual in EDTA tubes following a regular procedure. After centrifugation at 3000 g for 15 min at 4 °C, the plasma was aspirated and stored at -80 °C before use. Thirteen types of clinical information were collected in this cohort including age, bone mass index (BMI), procollagen I N-Terminal propeptide (PINP), β isomer of C-terminal telopeptide of type I (β.CTX), T-score of each part of the lumbar spine (L1-L4), total T-score, Calcium (Ca), Magnesium (Mg), Phosphorus (P), cholesterol (CHOL), triglyceride (TG), high-density lipo-protein (HDL) and low-density lipoprotein (LDL).

**Exosomes isolation and characterization**

Exosomes were isolated using ExoQuick kit purchased from System Biosciences. The isolation procedure followed instructions provided with the kit. In brief, the plasma was pretreated with a coagulation reagent (Thrombin Plasma prep for Exosome precipitation). Plasma was then centrifuged at 3000 g for 15 min to remove fine cells and debris. The supernatant was transferred to a sterile tube, and 63 μL of ExoQuick Exosome precipitation solution was added in every 250 μL of supernatant. The tube was refrigerated at 4 °C for 30 min before it being centrifuged at 1500 g for 30 min at room temperature. The supernatant was aspirated and centrifuged at 1500 g for 5 min, and the residual ExoQuick solvent was carefully removed. The precipitated white particles at the bottom contained exosomes. The obtained exosomes were resuspended using sterile PBS filtrate.

The size distribution of isolated exosomes was determined by nanoscale flow cytometry (nFCM) analysis, and the shape of exosomes were characterized by transmission electron microscope (TEM). The protocols were followed as described by previous report. Western blot was used to detect the Flotillin-1, CD9 and CD81 proteins on the surface of the exosomes.

**RNA extraction, library preparation and sRNA sequencing**

MiRNAs were extracted using the miRNeasy Mini Kit following standard procedures. The quality of RNAs was subsequently assessed using the Aglient 2100 Bioanalyzer. The library preparation involved the following brief steps: fragments selection, adapter ligation, reverse transcription, PCR amplification, purify PCR products, library quality control. Single-stranded PCR products are produced via denaturation, the reaction system and program for circularization were subsequently configured and set up. Single-stranded circle DNA molecules were replicated *via* rolling cycle amplification, and a DNA nanoball (DNB) which contain multiple copies of DNA was generated. It was subjected to sequencing on the MGISEQ-500 platform.

**MiRNAs quantification**

The sRNAnalyzer toolkit was used to perform quality control on raw data, which involved deleting sequencing adapters, trimming low-quality bases, and retaining reads with a length of 15nt-32nt. Alignment and identification of sRNAs were performed on the ‘processed.fa’ file obtained after filtering. We used the ‘align.pl’ module to compare the database, and all database information was configured in the ‘DB_config.conf’ file. The primary databases used for the analysis were miRbase v21.0 for miRNAs, piRbase v2.0 for piRNAs, snoRNAbase v1.0 for snoRNAs, and GtRNADB v1.0 for other noncoding RNAs.

**Risk factor control**

Several clinical variables may be potential risk factors except for t-score when developing biomarkers for PMOP, including age, blood biochemical markers, bone turnover markers, BMI, history of metabolic disease, estrogen levels, lifestyle, etc. Considering the convenience of collection, this study collected multiple clinical information, such as age, history of metabolic diseases, the concentration of bone turnover biomarkers, Ca^2+^, Mg^2+^, P^3-^, CHOL, TG, HDL, LDL in blood.

**Statistical analysis**

The reads count of miRNAs was used to perform differential expression analysis using ‘DESeq2’ R packages, with the thresholds set at *p* < 0.05, log_2_(FoldChange) > 0.6 based on the actual situation. Moreover, the normalization of raw reads using reads per million (RPM) was required for directly comparing differential expression levels among miRNAs.

The differentially expressed miRNAs (DE-miRNAs) were used as dependent variables to construct a lasso-logistical regression model following a minimalist model principle were obtained using the ‘glmnet’ R package. The risk score for each sample reflects their contribution to osteoporosis, the risk score model was constructed based on a linear combination of the expression level and the regression coefficient (β) from the lasso-logistical regression. The formula was as follows: risk score = expression of gene1 × β1 + expression of gene2 × β2 + … expression of gene(n) × βn.

T-test was also used to analyze the expression differences and the false discovery rate (FDR) was controlled for multiple comparisons. A significance level of *p* < 0.05 was considered significant. Detection ability of potential biomarkers was assessed by receiver operating characteristic (ROC) curves analysis, and the area under the curve (AUC) was also calculated. The ‘ComplexHeatmap’ and ‘ggplot2’ R packages were used for visualization of results.

**RT-qPCR analysis**

The selected plasma-derived exo-miRNAs were validated using the TaqMan Advanced miRNA Assays Protocol (Applied Biosystems). The TaqMan Advanced miRNA cDNA Synthesis Kit (Applied Biosystems, catalog no. A28007) was used to synthesize, adapt a 3′-poly(A) tail and a 5′-adaptor ligation to the miRNAs, then miRNA cDNA was synthesized using universal RT primers and amplified in miR-AMP step. The obtained cDNA was diluted 1 in 10 with RNase-free water prior to being applied in RT-qPCR. RT-qPCR reactions were run on a QuantStudio 6 Flex Real-Time PCR System (Applied Biosystems) using the TaqMan Fast Advanced Master Mix (Applied Biosystems, catalog no. 4444557) and specific TaqMan Advanced miRNA Assays (Applied Biosystems, catalog no. A25576), each reaction was performed in duplicate samples. We used hsa-miR-191-5p as internal references to measure the relative expression level of selected miRNAs using Delta-Delta Ct method.
