# Supplementary figures and images for: A plasma‐derived exosomal microRNA signature by small RNA sequencing for early detection of postmenopausal osteoporosis
Source: Clin Transl Med. 2024 Apr 1;14(4):e1637. doi: 10.1002/ctm2.1637 (PMC10983017; doi:10.1002/ctm2.1637)

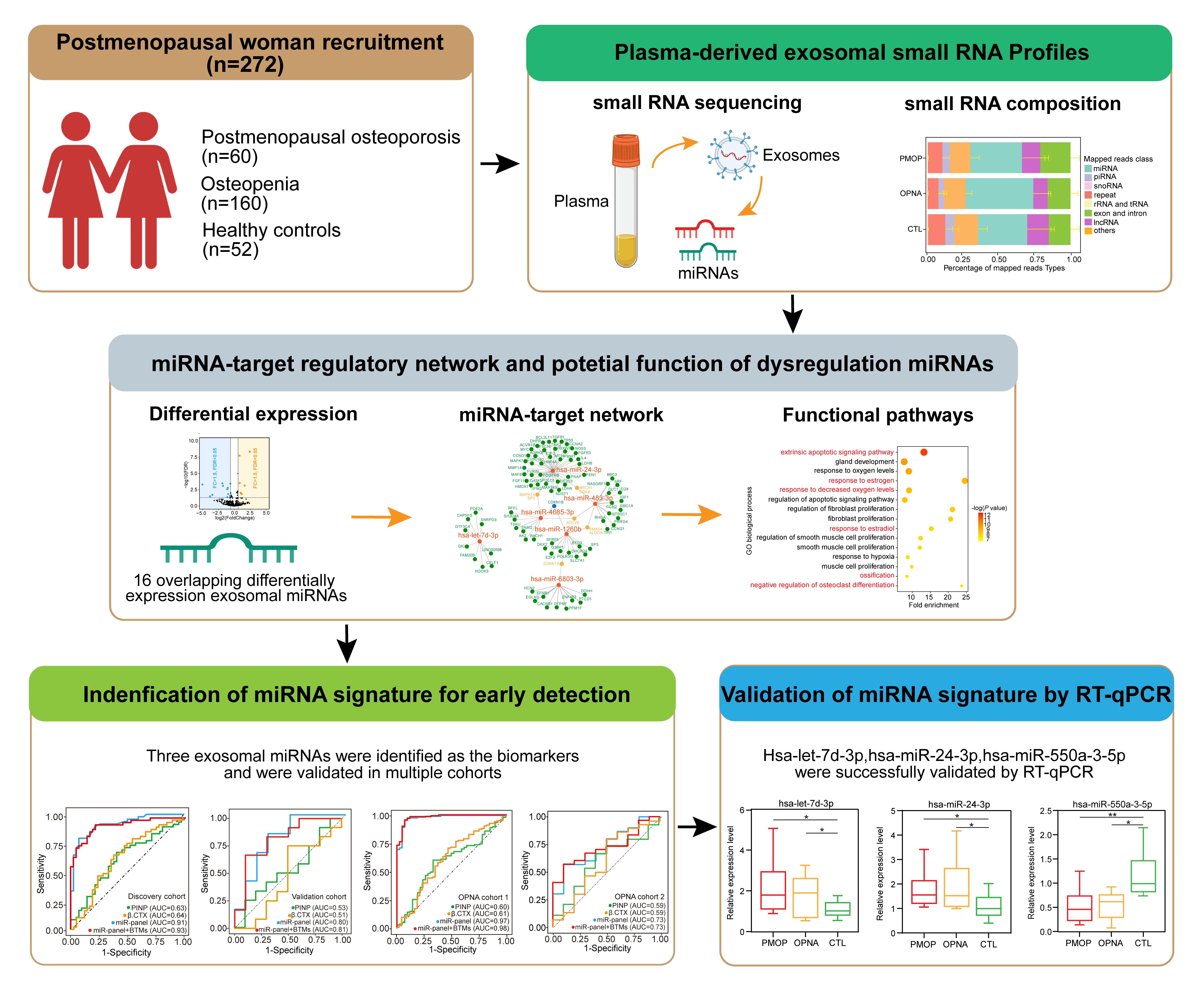

Supplement: Supplementary file 2 — Supporting Information [file CTM2-14-e1637-s006.png]

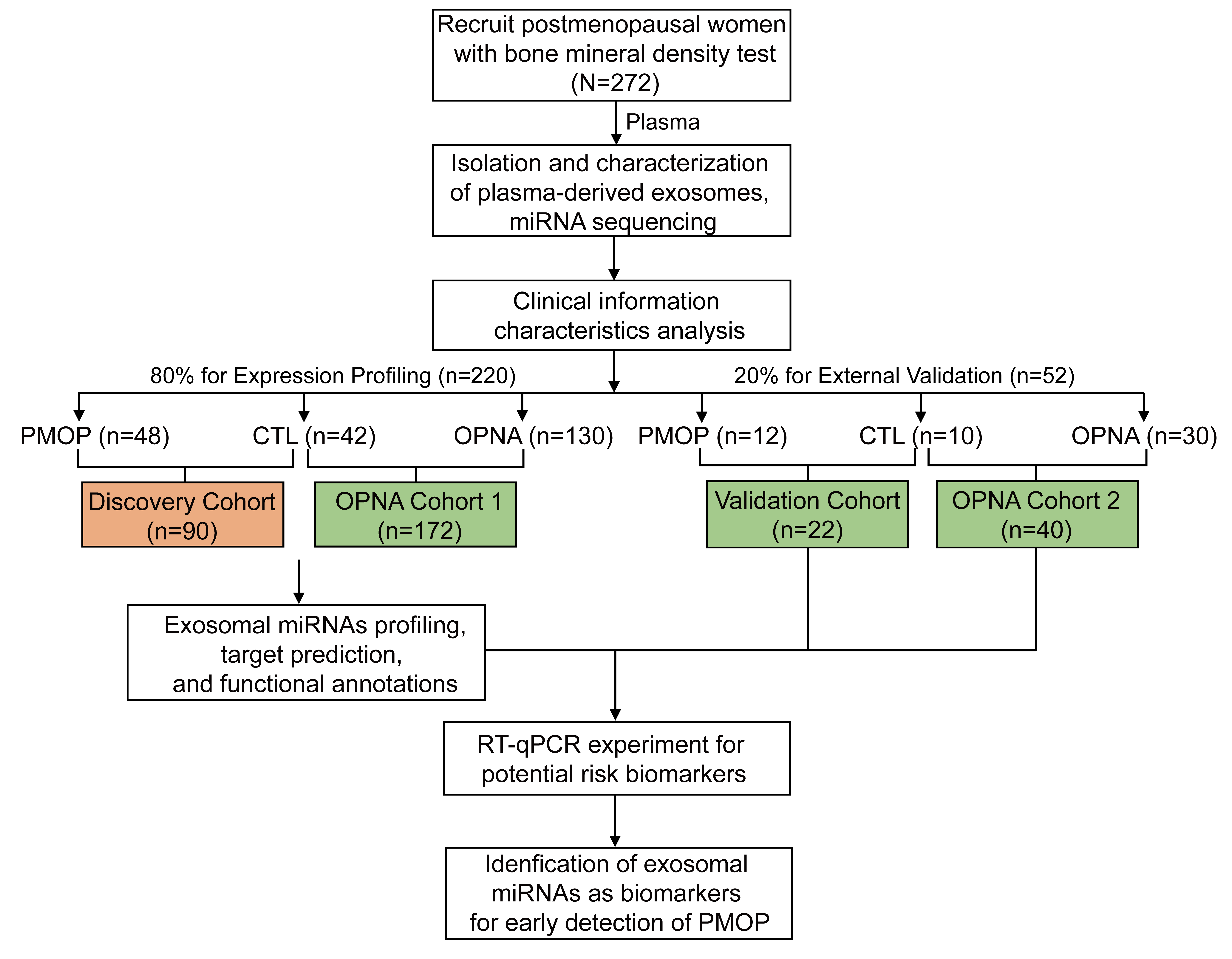

Supplement: Supplementary file 3 — Supporting Information [file CTM2-14-e1637-s011.png]

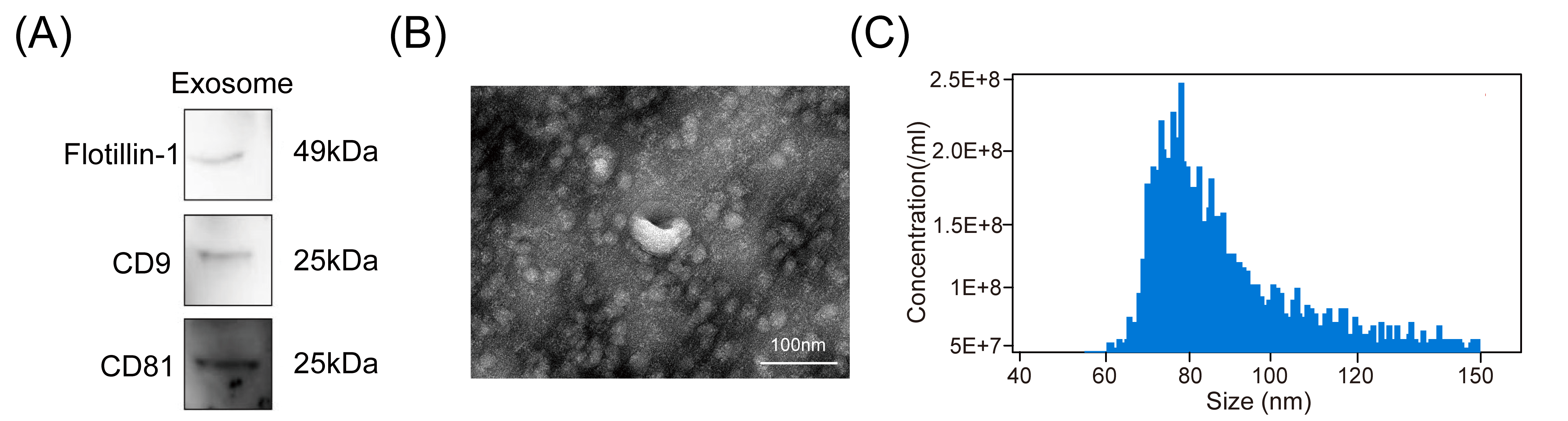

Supplement: Supplementary file 4 — Supporting Information [file CTM2-14-e1637-s002.png]

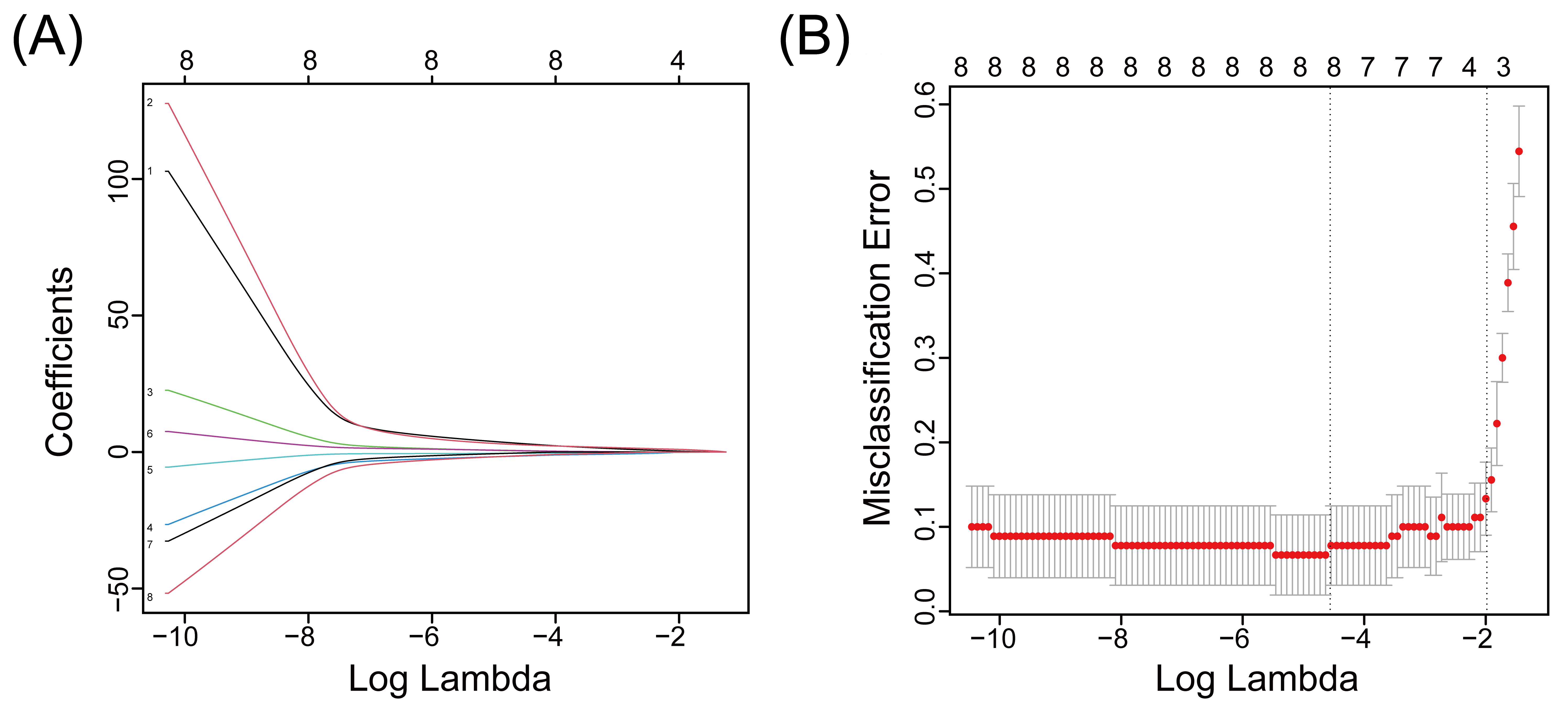

Supplement: Supplementary file 5 — Supporting Information [file CTM2-14-e1637-s015.png]

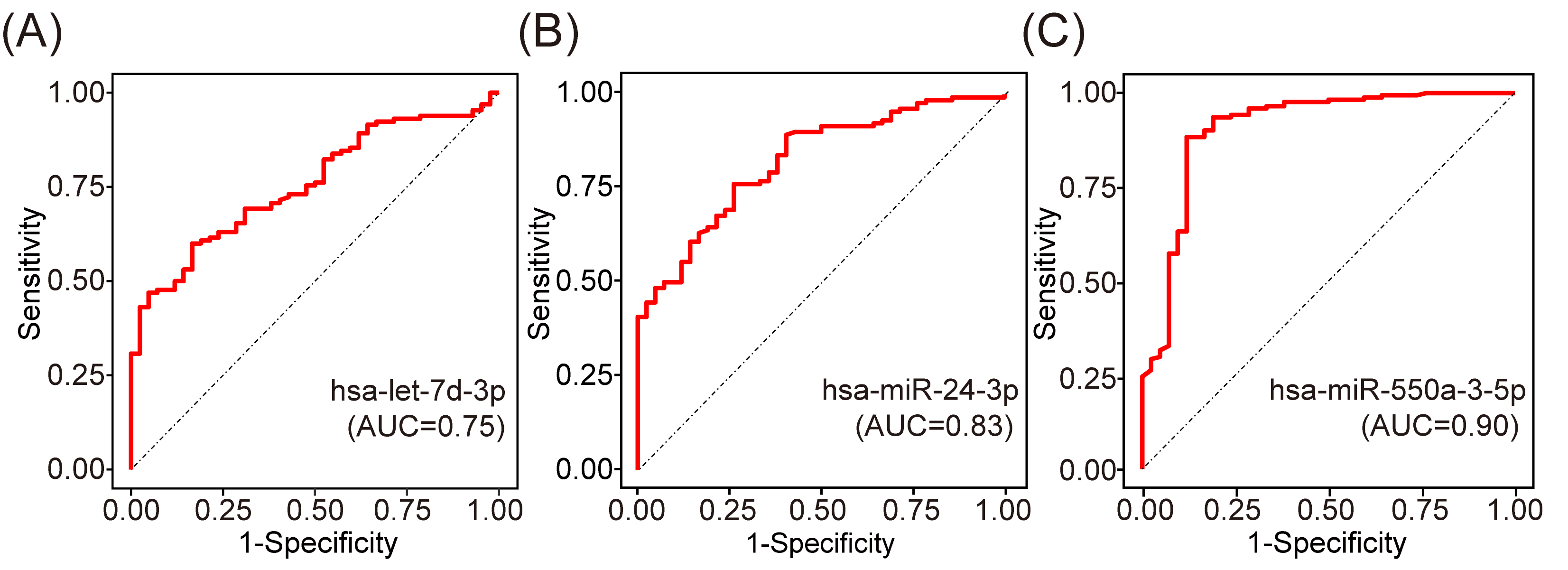

Supplement: Supplementary file 6 — Supporting Information [file CTM2-14-e1637-s010.png]

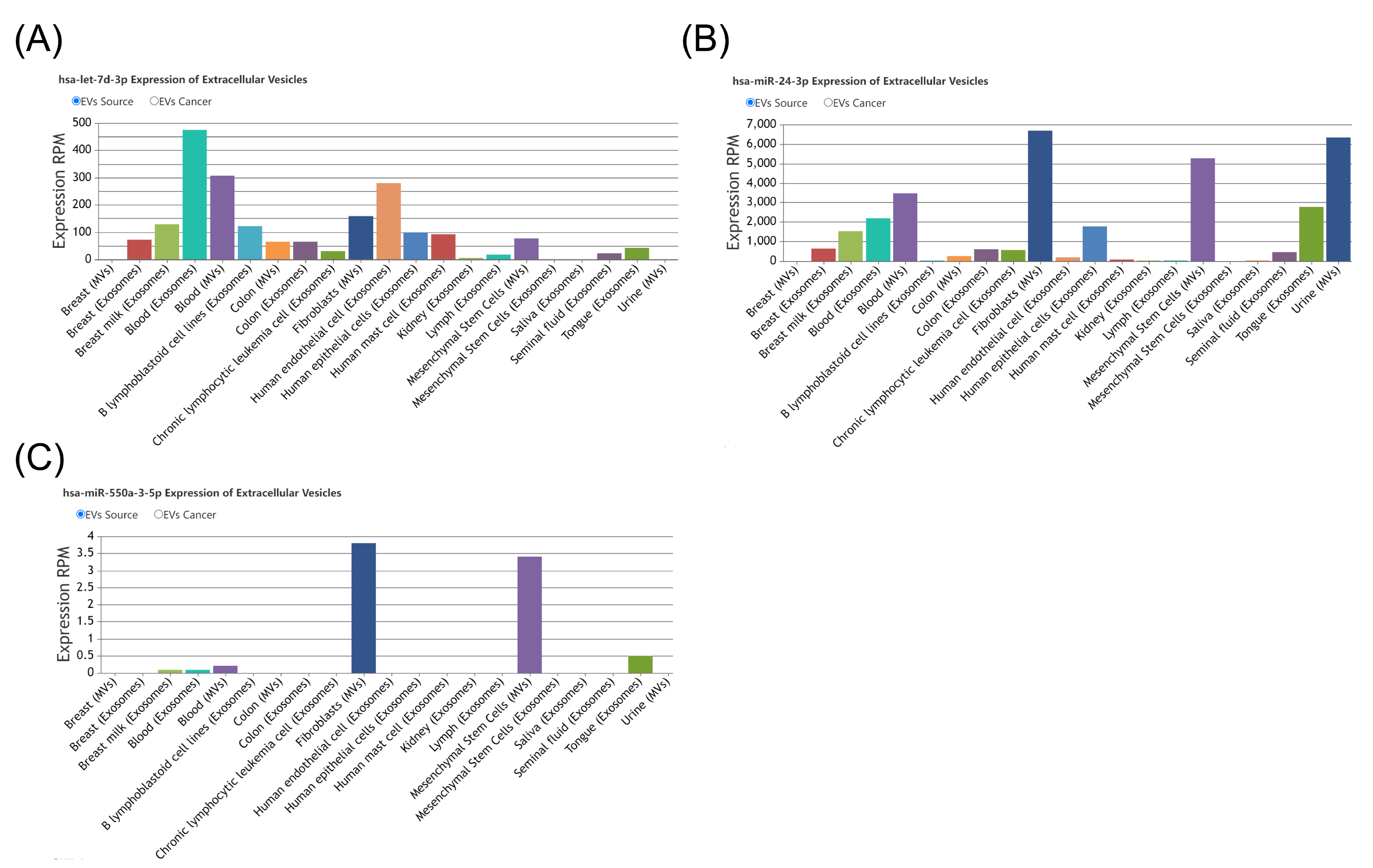

Supplement: Supplementary file 7 — Supporting Information [file CTM2-14-e1637-s022.png]

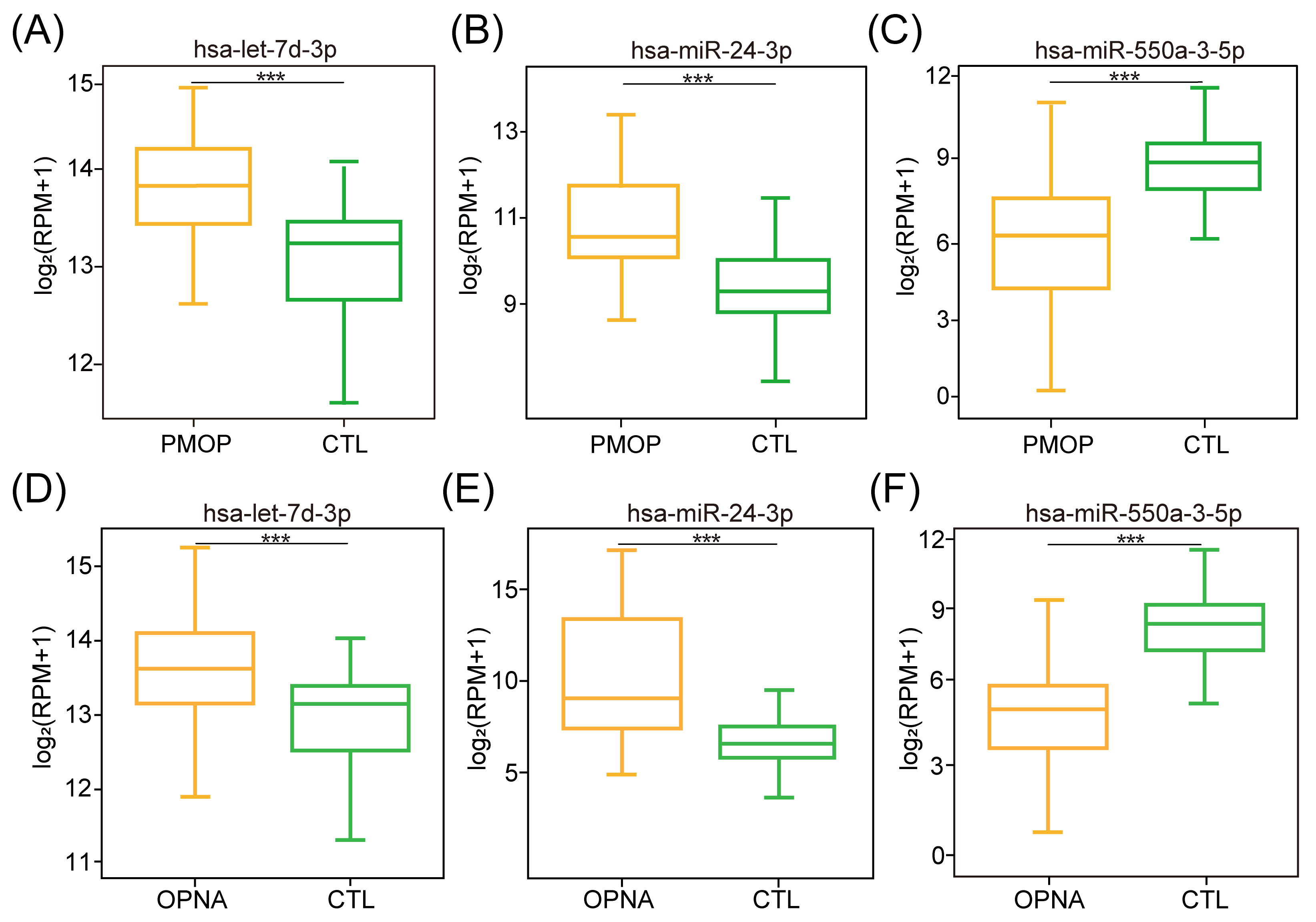

Supplement: Supplementary file 8 — Supporting Information [file CTM2-14-e1637-s019.png]
