# Supplementary material for: A plasma‐derived exosomal microRNA signature by small RNA sequencing for early detection of postmenopausal osteoporosis
Source: Clin Transl Med. 2024 Apr 1;14(4):e1637. doi: 10.1002/ctm2.1637 (PMC10983017; doi:10.1002/ctm2.1637)
Supplement: Supplementary file 22 — Supporting Information [file CTM2-14-e1637-s001.docx]

**Appendices**

**Supplementary figure legends**

**FIGURE S1** Workflow of this study.

**FIGURE S2** Schematic diagram showing the sample grouping information and the analysis procedure.

**FIGURE S3** Plasma-derived exosomes characterization. (A) Western blot analysis. (B) TEM imaging result. (C) The size distribution of plasma-derived exosomes.

**FIGURE S4** Screening of exosomal miRNAs as risk biomarker for PMOP. (A) Potential contribution of miRNAs to PMOP risk. (B) Cross validation to determine the optimal combination of miRNAs.

**FIGURE S5** The detection performance of the three exo-miRNAs in the OPNA cohort. The ROC curve for hsa-let-7d-3p (A), hsa-miR-24-3p (B), and hsa-miR-550a-3-5p (C), respectively in the OPNA cohort 1.

**FIGURE S6** The expression level of hsa-let-7d-3p (A), hsa-miR-24-3p (B), and hsa-miR-550a-3-5p (C) in different source tissues/cells.

**FIGURE S7** The relative expression level of the three exo-miRNAs in the PMOP, OPNA and CTL groups. The relative level of the hsa-let-7d-3p (A), hsa-miR-24-3p (B), and hsa-miR-550a-3-5p (C) between PMOP and CTL. The relative levels of the hsa-let-7d-3p (D), hsa-miR-24-3 (E), and hsa-miR-550a-3-5p (F) between OPNA and CTL. (* represents *p*<0.05, ** represents *p*<0.01, *** represents *p*<0.001).

**Supplementary tables**

**TABLE S1.** The clinical phenotypic information for all participants.

**TABLE S2.** Clinical characteristics of all participants enrolled in this study.

**TABLE S3.** Percentage of all clean reads mapped to various ncRNA database in the three groups.

**TABLE S4.** Identified exo-miRNAs in this study.

**TABLE S5.** The DE-miRNAs in this study.

**TABLE S6.** The target genes of selected exosomal DE-miRNAs.

**TABLE S7.** GO terms of the upregulated exosomal miRNAs targets.

**TABLE S8.** KEGG pathways of the upregulated exosomal miRNAs targets.

**TABLE S9.** GO terms of the downregulated exosomal miRNAs targets.

**TABLE S10.** KEGG pathways of the downregulated exosomal miRNAs targets.

**TABLE S11.** The detailed AUC values of multiple components (three exo-miRNAs, PINP, β.CTX and the combinations) in the discovery cohort.

**TABLE S12.** The detailed AUC values of multiple components (three exo-miRNAs, PINP, β.CTX and the combinations) in the multiple validation cohorts.

**TABLE S13.** Pearson coefficients between clinical information and risk score of miRNA panel.
